# Supplementary material for: Influence of the corticospinal tract wiring pattern on sensorimotor functional connectivity and clinical correlates of upper limb function in unilateral cerebral palsy
Source: Sci Rep. 2019 Jun 3;9:8230. doi: 10.1038/s41598-019-44728-9 (PMC6547689; doi:10.1038/s41598-019-44728-9)
Supplement: Supplementary file 1 — Supporting information [file 41598_2019_44728_MOESM1_ESM.pdf]

Influence of the corticospinal tract wiring pattern on sensorimotor functional connectivity and clinical correlates of upper limb function in unilateral cerebral palsy

Cristina Simon-Martinez*[1] Cristina.Simon@kuleuven.be

Ellen Jaspers[2] Ellen.Jaspers@hest.ethz.ch

Kaat Alaerts[1] Kaat.Alaerts@kuleuven.be

Els Ortibus[3] Els.Ortibus@uzleuven.be

Joshua Balsters[2,4] Joshua.Balsters@rhul.ac.uk

Lisa Mailleux[1] Lisa.Mailleux@kuleuven.be

Jeroen Blommaert[5] Jeroen.Blommaert@kuleuven.be

Charlotte Sleurs[5] Charlotte.Sleurs@kuleuven.be

Katrijn Klingels[1,6] Katrijn.Klingels@kuleuven.be

Frédéric Amant[5,7,8] Frederic.Amant@uzleuven.be

Anne Uyttebroeck[5] Anne.Uyttebroeck@uzleuven.be

Nicole Wenderoth[2] Nicole.Wenderoth@hest.ethz.ch

Hilde Feys[1] Hilde.Feys@kuleuven.be

Nicole Wenderoth and Hilde Feys should be considered joint senior authors.

[1] KU Leuven Department of Rehabilitation Sciences, Leuven, Belgium

[2] Neural Control of Movement Lab, Department of Health Sciences and Technology, ETH Zurich, Switzerland.

[3] KU Leuven Department of Development and Regeneration, Leuven, Belgium

[4] Department of Psychology, Royal Holloway University of London, Egham, United Kingdom

[5] KU Leuven Department of Oncology, Leuven, Belgium

[6] Rehabilitation Research Centre, Faculty of Rehabilitation Sciences, Hasselt University, Diepenbeek, Belgium

[7] Centre for Gynaecologic Oncology, Antoni van Leeuwenhoek, Amsterdam, Netherlands

[8] Centre for Gynaecologic Oncology, Amsterdam University Medical Centres, Amsterdam, Netherlands

*Corresponding author:

Cristina Simon-Martinez; cristina.simon@kuleuven.be; Phone number: 0032 483 72 94 17

Herestraat 49, box 1510

ORCID: 0000-0001-6694-6358

Supporting Information

Table S1. Descriptive demographic data of each cohort.

		TD cohort (n=60)	Contralateral CST (n=9)	Bilateral CST (n=6)	Ipsilateral CST (n=9)
**MACS levels**	n (%)				
I			7 (78)	0 (0)	1 (11)
II			1 (11)	5 (83)	5 (56)
III			1 (11)	1 (17)	3 (33)
**Age (years)**	mean (SD)	14.54 (4.80)	14.54 (4.18)	10.88 (3.41)	13.36 (5.11)
**Head motion (mean FD)**†	mean (SD)	0.26 (0.12)	0.31 (0.14)	0.47 (0.30)	0.25 (0.14)
**Sex**	n (%)				
Male		46 (77)	2 (22)	3 (50)	4 (44)
Female		14 (23)	7 (78)	3 (50)	5 (56)
**Dominant hand**	n (%)				
Right		54 (90)	2 (22)	5 (83)	6 (67)
Left		6 (10)	7 (78)	1 (17)	3 (33)

TD, typically developing; CST, corticospinal tract; MACS, Manual Ability Classification System; FD, frame wise displacement; SD, standard deviation. †Head motion (mean FD) was not different between the TD and the uCP cohorts ($p>0.05$).

Table S2. Correlation coefficients (Pearson's r (p-value)) between functional connectivity measures and UL motor function in the uCP cohort.

		**Bimanual performance (AHA)**	**Hand dexterity (JTHF test, log10)**	**Grip strength (log10)**
**Intra FC Non-dom**	**M1-PMd**	0.01 (0.97)	0.06 (0.77)	-0.14 (0.50)
	**M1-PMv**	0.00 (0.99)	0.07 (0.74)	-0.09 (0.69)
	**M1-S1**	0.20 (0.33)	-0.17 (0.41)	-0.14 (0.49)
**Intra FC Dom**	**M1-PMd**	0.17 (0.42)	-0.06 (0.77)	-0.17 (0.42)
	**M1-PMv**	-0.07 (0.75)	0.17 (0.42)	0.03 (0.88)
	**M1-S1**	-0.21 (0.32)	0.10 (0.65)	0.00 (0.99)
**Laterality index (intrahemispheric imbalance)**		-0.29 (0.15)	0.15 (0.46)	0.26 (0.21)
**Intra FC Dom**	**M1-(PO-SMG)**	-0.06 (0.77)	0.23 (0.18)	0.07 (0.72)
**Inter FC Non-dom → Dom**	**M1-PMd**	-0.06 (0.78)	-0.02 (0.91)	0.03 (0.89)
	**M1-PMv**	0.03 (0.89)	-0.07 (0.73)	-0.06 (0.78)
	**M1-S1**	0.01 (0.98)	-0.17 (0.42)	-0.06 (0.78)
	**M1-SMA**	-0.30 (0.15)	0.28 (0.18)	0.02 (0.92)
**Inter FC Dom → Non-dom**	**M1-PMd**	0.29 (0.15)	-0.20 (0.33)	-0.24 (0.25)
	**M1-PMv**	-0.13 (0.54)	0.09 (0.67)	0.01 (0.95)
	**M1-S1**	0.35 (0.09)	-0.39 (0.05)	-0.36 (0.08)
	**M1-SMA**	-0.12 (0.57)	0.30 (0.15)	-0.21 (0.32)
**Inter**	**M1-M1**	-0.06 (0.77)	0.28 (0.18)	0.07 (0.72)

FC, functional connectivity; Non-dom, non-dominant hemisphere; Dom, dominant hemisphere; M1, primary motor cortex; PMd, dorsal stream of the premotor cortex; PMv, ventral stream of the premotor cortex; S1, primary sensory cortex; SMA, supplementary motor area; PO, parietal operculum; SPM, supramarginal gyrus; AHA, assisting hand assessment; JTHF, Jebsen-Taylor hand function.

Table S3. MNI coordinates of the ROIs included in the analysis.

	Non-dominant hemisphere			Dominant hemisphere		
	**x**	**y**	**z**	**x**	**y**	**z**
**PMd**	-30	-7	63	30	-7	63
**PMv**	-51	4	24	51	4	24
**M1**	-37	-25	62	37	-25	62
**S1**	-40	-27	53	40	-27	53

	Midline structure (x, y, z)		
**SMA proper**	0	-10	59

SMA, supplementary motor area; PMd, premotor cortex dorsal part; PMv, premotor cortex ventral part; M1, primary motor cortex; S1, primary sensory cortex.
